# LC-ESI/LTQ-Orbitrap-MS Based Metabolomics in Evaluation of Bitter Taste of *Arbutus unedo* Honey

**DOI:** 10.3390/molecules26092765

**Published:** 2021-05-08

**Authors:** Paola Montoro, Gilda D’Urso, Adam Kowalczyk, Carlo Ignazio Giovanni Tuberoso

**Affiliations:** 1 Department of Pharmacy, University of Salerno, via Giovanni Paolo II, 132, 84084 Fisciano, Italy; pmontoro@unisa.it (P.M.); gidurso@unisa.it (G.D.); 2 Department of Pharmacognosy and Herbal Medicines, Wrocław Medical University, ul. Borowska, 211, 50-556 Wrocław, Poland; adam.kowalczyk@umed.wroc.pl; 3 Department of Life and Environmental Sciences, University of Cagliari, University Campus, S.P. Monserrato-Sestu km 0.700, 09042 Monserrato, Italy

**Keywords:** unedone, bitter taste, strawberry tree honey, LC-ESI/LTQ-Orbitrap-MS, PCA, PLS

## Abstract

Strawberry tree honey is a high-value honey from the Mediterranean area and it is characterised by a typical bitter taste. To possibly identify the secondary metabolites responsible for the bitter taste, the honey was fractionated on a C18 column and the individual fractions were subjected to sensory analysis and then analysed by liquid chromatography coupled with high-resolution tandem mass spectrometry in negative ion mode, using a mass spectrometer with an electrospray source coupled to a hybrid high resolution mass analyser (LC-ESI/LTQ-Orbitrap-MS). A chemometric model obtained by preliminary principal component analysis (PCA) of LC-ESI/LTQ-Orbitrap-MS data allowed the identification of the fractions that caused the perception of bitterness. Subsequently, a partial least squares (PLS) regression model was built. The studies carried out with multivariate analysis showed that unedone (2-(1,2-dihydroxypropyl)-4,4,8-trimethyl-1-oxaspiro [2.5] oct-7-en-6-one) can be considered responsible for the bitter taste of strawberry tree honey. Confirmation of the bitter taste of unedone was obtained by sensory evaluation of a pure standard, allowing it to be added to the list of natural compounds responsible for giving the sensation of bitterness to humans.

## 1. Introduction

Strawberry tree (*Arbutus unedo* L.) (ST) honey is a peculiar bitter tasting honey produced in the area of the Mediterranean basin. This highly valuable honey is well recognised for its antioxidant [1,2,3], anti-inflammatory, and antimicrobial activities [4], and shows anti-xanthine oxidase and antityrosinase activities [5]. Several studies have been performed to characterise the chemical composition of ST honey, which is characterised by phenolic compounds (hydroxy derivatives of benzoic and cinnamic acids, and flavonoids), isoprenoids, and free amino acids [6,7]. Homogentisic acid (2,5-dihydroxyphenylacetic, HGA), unedone (2-(1,2-dihydroxypropyl)-4,8,8-trimethyl-1-oxaspiro [2.5]oct-4-en-6-one), (±)-2-*cis*, 4-*trans*-abscisic acid (*c*,*t*-ABA), and (±)-2-*trans*, 4-*trans*-abscisic acid (*t*,*t*-ABA) have been considered as chemical markers of the botanical origin of ST honey [8,9].

Although ST honey has been investigated for its chemical composition, no studies have been reported as regards the possible compounds responsible for its bitter taste so far. Arbutin, the glucosylated form of hydroquinone, has been suggested as possibly responsible for the bitter taste of ST honey [4]. However, although the bitter taste perception of arbutin is very strong [10] and this compound can be found abundantly in *A. unedo* plant, its presence in ST honey is variable, often insignificant, and sometimes it is totally absent [4]. Thus, the involvement of arbutin in the bitter taste of ST honey is very unlikely. Literature data indicate many plant-derived bitter-tasting compounds [11,12]. They can be represented by alkaloids, terpenoids, phenols, amino acids, and peptides, and they activate the bitter taste receptors (T2R). Comparison of the known natural compounds responsible for the bitter taste with the molecules reported in the literature for ST honey did not make it possible to speculate which compound was responsible for the typical bitter taste of this honey.

Given the above, investigation of ST honey using the metabolomic approach could help in detecting the compounds responsible for the bitter taste in ST honey. Metabolomics, as an emerging discipline of omics science, is a valid tool for the characterization of complex biological samples as it allows the production of a molecular fingerprint for samples by using innovative analytical techniques, such as mass spectrometry (MS) and nuclear magnetic resonance (NMR) [13,14]. Particularly, liquid chromatography-high resolution mass spectrometry metabolic profiling has begun to be used to discover possible markers in foods, especially those most likely responsible for the biological activities [15,16,17,18].

The aim of this study was to develop an LC-ESI/LTQ-Orbitrap-MS based metabolomic approach in the evaluation of the compounds responsible for the bitter taste of *Arbutus unedo* honey. To this purpose, polar compounds from strawberry tree honey were separated on a column containing C18 resin and the different fractions obtained were submitted to sensory analysis and investigated by high resolution mass spectrometry ((HR) LC-ESI/ LTQ-Orbitrap-MS, (HR) LC-ESI/LTQ-Orbitrap-MS/MS) and by HPLC-DAD. In addition, principal component analysis (PCA) of LC-ESI/LTQ-Orbitrap-MS data and a partial least squares (PLS) regression model were used to identify compounds responsible for the bitter taste. Finally, sensorial analysis on targeted pure compounds was performed to confirm the molecule responsible for the bitter taste in strawberry tree honey.

## 2. Results and Discussion

### 2.1. LC-ESI/LTQ-Orbitrap-MS

**Figure 1:**
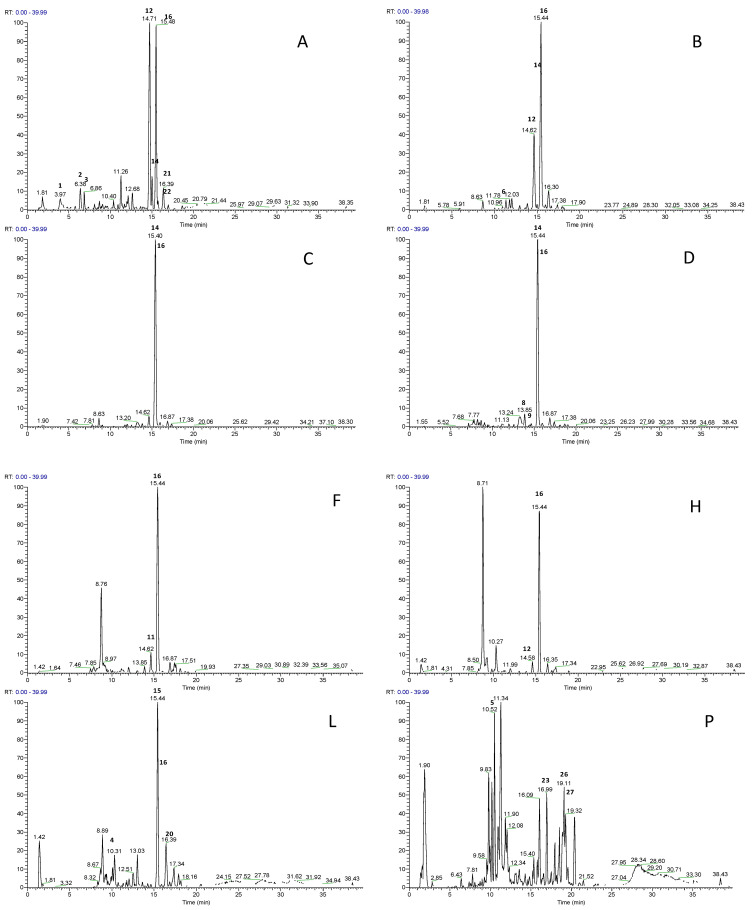

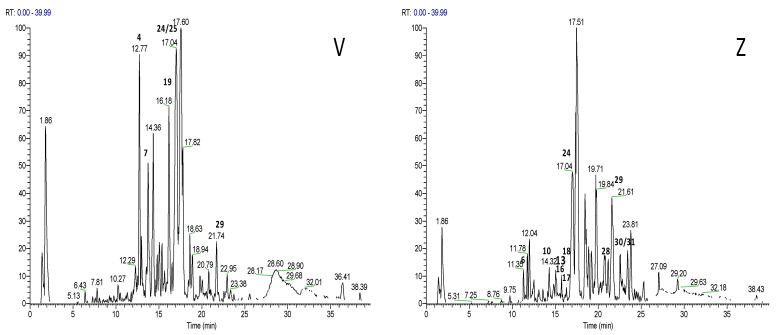
LC-ESI-LTQ-Orbitrap-MS base peak profiles of ten representative strawberry tree honey fractions (A, B, C, D, F, H, L, P, V, Z). Base peak intensity was fixed at NL 7.02E^6^ for all the chromatograms.

The 21 fractions obtained from the fractioning on C18 column were tasted to evaluate the impact of bitterness and were classified by comparison with quinine solutions (see Section 3.3) in five groups of bitter perception (from 0 to 4) as follows: no bitter taste (0, fractions A, B and C), barely detectable (1, fractions D, E, M, N, O, Q, R, S, T and U), weak perception (2, fractions F and V), moderate perception of bitterness (3, fractions G, H, I, L and P), and strong perception (4, fraction Z) (Table S1). Fraction E showed an LC-MS profile similar to fraction D, fractions M, N, O, Q, R, S, T, and U showed profiles similar to fraction P, while fractions G and I showed profiles similar to fraction F; therefore, only representative fractions with different profiling characteristics are shown in Figure 1.

The accurate mass measurement (ppm ≤ 5) and the MS/MS experiments in negative ionization mode, together with the comparison with the data present in the literature and databases, such as KNApSAcK [19], allowed to identify 29 chemical constituents reported in Table 1. Analysis of the samples was performed also in positive ion mode, but the better answer from the instrument was in negative ion mode. For this reason, the study was carried out using the negative polarity. An identification level was assigned to each sample, referring to the usual four levels of identification in metabolomic analyses [20].

Compound **2** showed an ion [M − H]^−^ at *m*/*z* 167.0346 corresponding to the molecular formula C_8_H_8_O_4_,. The compound was identified as a homogentisic acid, already reported in ST honey [8] and considered one of the marker compounds to evaluate the botanical origin of this honey along with *c*,*t*-ABA (**10**) and *t*,*t*-ABA (**14**) [9]. Compounds **2**, **10** and **14** were found to be present in fractions A, B, and C, the sweetest fractions. Compound **3** and compound **22** have already been reported in the literature [22]. In particular, compound **3** showed an ion [M − H]^−^ at *m*/*z* 481.1310 corresponding to the molecular formula C_22_H_26_O_12_ and was identified as arbutin peracetate, a compound present in the leaves of *A. unedo* [22]. Compound **22** showed an ion [M − H]^−^ at *m*/*z* 177.0917 corresponding to the molecular formula C_11_H_14_O_2_ and has been identified as *tert*-butylbenzoic acid [36]. Compounds **5**, **13**, and **24** have been reported in Algerian origin honey and have been identified as camphoric acid, di-*tert*-butyl-benzoquinone and di-*tert*-butyl-benzoquinone isomer, respectively [24]. Compounds **6**, **8**, **12**–**13**, **15**–**21**, **23**–**29** have already been reported to be present in different types of honey. Compound **6** showed an ion [M − H]^−^ at *m*/*z* 301.1798 corresponding to the formula C_19_H_26_O_3_, and was identified as allethrin, an insecticide already found in other honeys [25]. Compounds **8**, **28**, and **29** are phenolic compounds and were identified, respectively, as sakuranin [27], chrysin [40], and kurarinone [41], and were found to be present in the more bitter fraction Z. Compound **12** was identified as 2-hydroxyisophorone already reported in the ST honey of Sardinia and mostly present in sweet fractions. Finally, Compound **15** showed an ion [M − H]^−^ at *m*/*z* 239.0910 corresponding to the molecular formula C_13_H_20_O_4_. It was identified as unedone, a compound previously reported in strawberry tree honey [9] and more abundantly present in the bitter fraction Z. Thus, Compound **15** could be one of the metabolites particularly responsible for the bitter taste, along with Compounds **8** and **29**. Interestingly, no arbutin was detected in any of the ST fractions.

### 2.2. Untargeted Metabolomic Analysis of Strawberry Tree Honey Fractions

**Figure 2:**
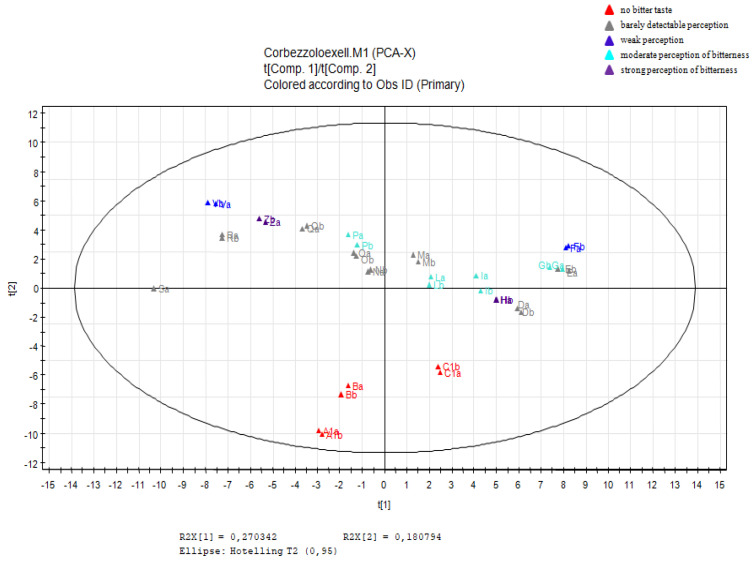
Principal component analysis: score scatter plot obtained from the untargeted analysis of the A-Z fractions.

**Figure 3:**
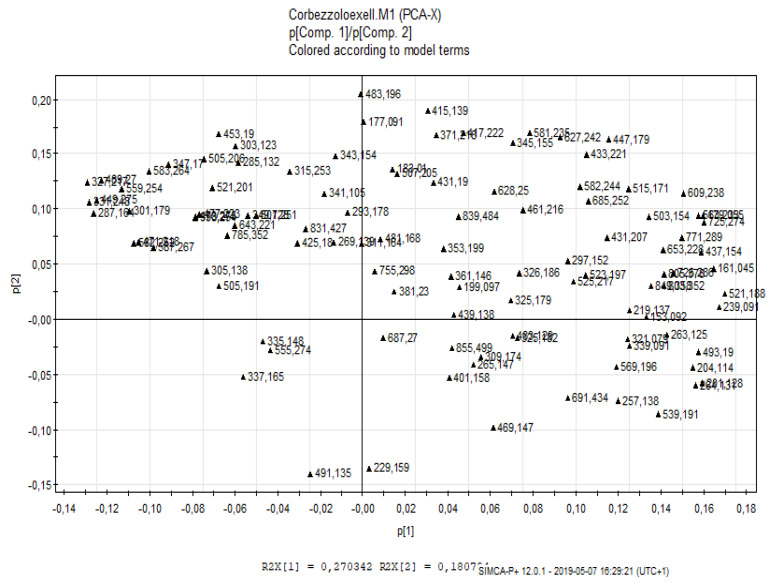
Principal component analysis: loading scatter plot obtained from the untargeted analysis of the A–Z fractions.

For the untargeted approach, the LC-ESI/LTQ-Orbitrap-MS chromatograms were pre-treated using the free software Mzmine [42], to compensate for changes in retention time and *m*/*z* ratio values between the chromatograms. The pre-treated chromatograms were exported as a data matrix, with the rows relative to the individual samples and the columns relative to the integrated and normalised peak areas obtained through LC-ESI/LTQ-Orbitrap-MS. The numerical values attributed to the variables were pre-treated through logarithmic transformation. Data transformation is intended to remove unwanted systematic behaviour. The exploratory analysis of the samples in terms of similarity or differences was performed using the PCA projection method. The score scatter plot obtained from PCA is shown in Figure 2. The graph shows good discrimination between the sweet fractions coloured in red at the bottom of the plot and the more bitter fractions located in the upper part of the plot. Therefore, it is the second principal component that has a more pronounced influence on the spatial distribution of the samples in the score scatter plot. PCA remains an unsupervised technique, which, therefore, cannot have predictive value and can only provide us preliminary information on the biochemical markers underlying the classification, by reading the corresponding loading scatter plot (Figure 3), in which the variables corresponding to the *m*/*z* values are shown. In particular, variables that contribute most to the differentiation of the samples in the score scatter plot and to their location in a specific area of the space can be highlighted.

To classify the samples and to understand which metabolites were most responsible for the bitter taste, the data were statistically processed through another projection technique, the partial least square (PLS) analysis. When the system under consideration is described by a data table and one or more single variable (Y) and the question is what relationship exists between the data block and the single variable (Y), the multivariate technique that is applied is the projection to latent structures by PLS. Therefore, the PLS projection aims to find linear relationships and then to build a plot of the variables that can explain this linear relationship. The model built from the data matrix obtained from the variables, and subsequently transformed and scaled (X), correlated with the Y relative to the perception of bitter taste, was then validated as suggested by Schievano et al. [43] and Stocchero [44], through the analysis of Q2, whose value was higher than 0.5, and through the permutation test, a test through which it is possible to evaluate the randomness and the presence of overfitting in the model.

**Figure 4:**
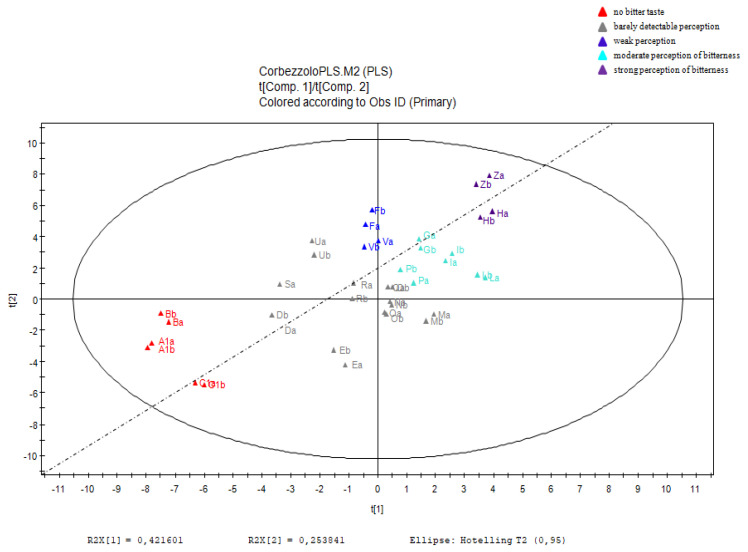
Partial least square: score scatter plot. Untargeted analysis of A–Z fractions.

**Figure 5:**
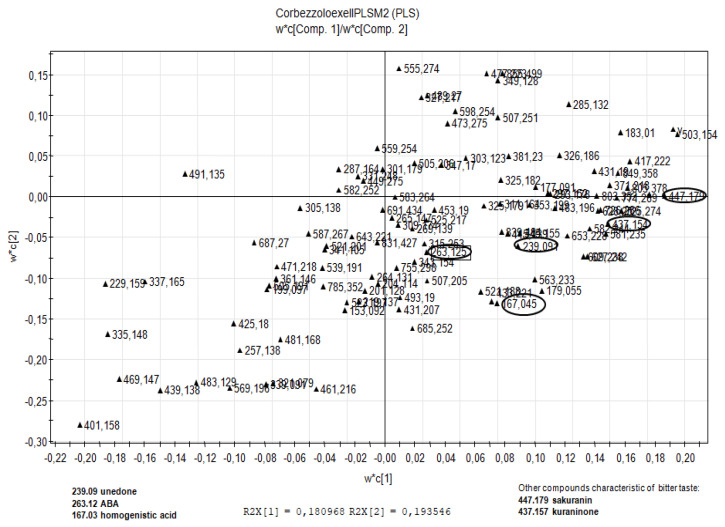
Partial least squares: loading scatter plot. Untargeted analysis of A–Z fractions.

As regards the fractions of ST honey, in the space built between t1 and t2 the samples (observations) seem to line up according to a linear relationship that sees in the positions at the top right of the plot the fractions capable of giving the most decisive sensation of bitter taste, and in the lower left the fractions capable of giving the most decisive sensation of sweet taste. The linear relationship can be seen in the score scatter plot (Figure 4). From the analysis of the loading scatter plots (Figure 5), in addition to the construction of a w*c plot, it is possible to evaluate the effect of the variables of the X block on the Y response and, in the specific case, determine the values of *m*/*z* observed in the chemical profiling that cause the fractions to have more or less pronounced Y responses (perception of the bitter taste).

From this analysis, the variables found in the loading plot area closest to the Y variable are those that have the highest positive coefficient in the model. From the observation of the LSP, it seems that the influence of *t*,*t*-ABA, *c*,*t*-ABA, and homogentisic acid, described as floral markers of ST honey [9], is decidedly significant, being positioned in the central part of the plot and at a fair distance from the variable Y. Other compounds appear to be relevant in the upper right quadrant of the loading scatter plot, namely unedone (*m*/*z* 239.0910), sakuranin (*m*/*z* 447.1277), and kurarinone (*m*/*z* 437.1952).

### 2.3. Quantitative Analysis of Isoprenoid Compounds by HPLC-DAD

(HR) LC-ESI/LTQ-Orbitrap-MS analyses allowed the identification of the four typical ST honey floral markers (homogentisic acid, *c*,*t*-ABA, *t*,*t*-ABA, and unedone), and thus quantitative HPLC-DAD analysis was used to evaluate whether their variability in the different fractions was connected with the perception of the bitter taste. Homogentisic acid was excluded from this evaluation because it was known that the compound does not have a bitter taste [8]. Table 2 shows the average content of the single isoprenoid compounds (± standard deviation) in the different fractions and expressed as mg of the active ingredient in 1 g of dry fraction. As shown in Table 2, the floral markers *c,t*-ABA and *t*,*t*-ABA are mainly found in the fractions characterised by a less bitter taste, while unedone has been separated mainly in the Z fraction, which was that represented by a more intense sensation of bitterness (Figure S1).

Unedone, an epoxidised derivative of abscisic acid, seems to play a significant role in the bitter taste of ST honey, being positioned in the upper right quadrant of the loading scatter plot. For this reason, a commercial standard of unedone was submitted to sensory analysis. Interestingly, unedone was found to be bitter, and a 300 mg/L solution of unedone gave a level of bitter sensation similar to 10 mg/L quinine. Similarly, *c*,*t*-ABA and *t*,*t*-ABA were submitted to sensory analysis, but no bitter taste was detected for up to 1000 mg/L solutions. Pydi et al. [45] investigated several abscisic acid (ABA) precursors and metabolites on the T2R4 receptor. It was observed that the structure deeply affects the bitter taste perception. For instance, ABA acts as an antagonist for T2R4, while xanthoxin is an agonist. Interestingly, both unedone and xanthoxin present an epoxide structure. Thus, it can be speculated that unedone acts as an agonist on T2R4 receptor.

In addition to unedone, two other compounds of a flavonoid nature present in very low quantities in ST honey fraction Z were also worthy of interest, namely sakuranin and kurarinone. Indeed, several compounds of flavonoid nature show the property of inducing the perception of bitterness [11]; thus, sakuranin and kurarinone are presumed to be potential sensory biomarkers. Sakuranin is a flavanone, a glucosylated derivative of sakuranetin. Unfortunately, no pure standard of sakuranin was found from suppliers of chemicals, so it was not possible to evaluate its involvement in the bitter perception. However, sakuranetin, the aglycone of sakuranin, was submitted to sensory analysis and no bitter taste was detected for this compound up to 1000 mg/L solutions. Sakuranin, like other flavanones, shows inhibitory activity against acetylcholinesterases [46], enzymes involved in the typical neurodegeneration of Alzheimer’s disease. Finally, a pure standard of kurarinone was submitted to sensory analysis and no bitter taste was detected for up to 1000 mg/L solutions also for this flavonoid. Kurarinone has shown good anti-tumour activity against non-small cell lung cancer (NSCLC). The activity has been demonstrated both in vitro and in vivo and appears to be related to the induction of apoptosis in A549 cells [47]. Another property attributed to kurarinone is its antifibrotic activity in the treatment of hepatitis B [48].

To conclude, unedone can be considered responsible for the bitter taste of *A. unedo* honey due to its constant and abundant presence already reported in ST honey (30–50 mg/kg) [9,49], although other natural compounds can modulate its bitter perception in this unifloral honey.

This developed approach can be useful for future studies on other honey samples to detect specific markers and for food quality control.

## 3. Materials and Methods

### 3.1. Chemicals

All the used chemicals were of analytical grade. Standard of homogentistic, acid (±)-2-*cis*, 4-*trans*-abscisic acid, kurarinone, sakuranetin, and quinine sulphate were purchased from Sigma–Aldrich (Milan, Italy). (±)-2-*trans*, 4-*trans*-abscisic acid was purchased from A.G. Scientific, Inc (San Diego, CA, USA). Unedone was purchased from Chem Faces Biochemical Co., Ltd. (Wuhan, China). Methanol, acetonitrile, phosphoric acid 85% *w*/*w*, and absolute ethanol were obtained from Sigma–Aldrich (Milan, Italy). Acetonitrile, water, and formic acid of LC-MS grade were purchased from Merck (Darmstadt, Germany). Ultrapure water (18 MΩ cm) was obtained with Milli-Q Advantage A10 System apparatus (Millipore, Milan, Italy).

### 3.2. Strawberry Tree Honey and Preparation of the Hydrophilic Fractions

Strawberry tree (ST) honey was produced in Sardinia (Italy) in 2018. Unifloral origin was verified by melissopalinological analysis, sensorial evaluation, and LC-DAD evaluation of the typical markers (homogentisic acid, unedone and the two isomers of abscisic acid) [9].

Hydrophilic fractions (HF) of the honey were prepared by dissolving 500 g of the honey in 500 mL of water acidified at pH 4.5 with HCl. The solution was poured on a chromatographic column filled with C18 resin (Sigma–Aldrich) previously activated with ethanol and equilibrated with water. The column charged with honey was washed with water and all the eluted solutions were discharged because no bitter taste was perceived. Elution of the fractions was obtained with increasing quantities of ethanol (H_2_O: EtOH in the ratios 95:5, 90:10, 80:20, 70:30, 50:50, 0:100 *v*/*v*). In total, 21 fraction were obtained and bitterness was evaluated by testing the solution after ethanol removal by Rotavapor^®^.

### 3.3. Sensory Analysis

A pre-trained sensory panel (5 judges) was used to determine the taste of both the fractions from the ST honey and pure compounds. Panellists provided written consent prior to participating indicating that they were not allergic to ST honey (included its main known compounds) or quinine. For establishing the level of perceptions of bitterness or sweetness, solutions of quinine and sucrose were prepared in water at six concentrations in the range 5 to 500 mg/L for quinine and 2 to 200 g/L for sucrose. Recognition threshold was performed according to testing samples in rank order (ISO 8587:2006) and was fixed at 5 mg quinine /L for bitterness and at 5 g sucrose /L for sweetness. For the sensory evaluation of the fractions from the ST honey, a scoring scale graded on a 5-point scale of bitterness perception was used: (0) no bitter taste; (1) barely detectable; (2) weak; (3) moderate; and (4) strong. Both (5)-point scale of bitterness perception and unedone level of bitterness (prepared at 300 mg/L in water) were established by comparison with the six concentrations of quinine. The samples were equilibrated to room temperature (20 °C ± 1) and the analysis was done during daylight. 1 mL of standard solution at proper concentration was applied to the upper surface of the tongue for 15 s then the test solution was expectorated.

### 3.4. LC-MS/MS Analysis

The electrospray ionisation (ESI) source of a Thermo Scientific LTQ-Orbitrap XL (Thermo Fisher Scientific, Dreieich, Germany) mass spectrometer was tuned in negative ion mode with a standard solution of kaempferol-3-*O*-glucoside (l µg/mL) introduced at a flow rate of 10 µL/min by a syringe pump. Calibration of the Orbitrap analyser was performed using the standard LTQ calibration mixture composed of caffeine and the peptide MRFA (calibration solution purchased from the manufacturer), dissolved in 50: 50% *v*/*v* water/acetonitrile solution.

Resolution for the Orbitrap mass analyser was set at 30,000. The mass spectrometric spectra were acquired by full range acquisition covering *m*/*z* 120–1400 in LC-MS. The study of the fragmentations was carried out using the data dependent scan experiment mode, by which the most intense [M-H] ions were selected during the LC-MS analysis. The data recorded were processed with Xcalibur 2.0 software (Thermo Fisher Scientific, Dreieich, Germany).

LC/ESI/LIT Orbitrap MS was performed using a Thermo Scientific liquid chromatography system consisting of a quaternary Accela 600 pump and an Accela autosampler, connected to a hybrid linear ion trap (IT) Orbitrap mass spectrometer (Thermo Scientific). LC-ESI-Orbitrap-MS analyses were performed using a Phenomenex Luna (150 mm × 2.1 mm particle size 5 µm) column, eluted with water containing 0.1% formic acid (solvent A) and acetonitrile containing 0.1% formic acid (solvent B). A linear gradient program at a flow rate of 0.200 mL/min was used: 0–35 min, from 5 to 95% (B); then back to 5% (B) for another 5 min. Honey fractions were dissolved in water with a concentration of 1 mg/mL and 5 µL of each samples was injected. The ESI source and MS parameters were the same as those used by D’Urso et al. [50]: capillary voltage: −48 V; tube lens voltage: −176.47 V; capillary temperature: 280 °C; sheath and auxiliary gas flow (N_2_), 15 and 5; sweep gas: 0; spray voltage: 5.

### 3.5. Quantitative Analysis by HPLC-DAD

Quantitative analysis of the strawberry tree honey markers was carried out by a modified HPLC-UV method from Tuberoso et al. [2,9] using an Agilent 1260 Infinity system (Agilent Technologies, Palo Alto, CA, USA), equipped with a binary pump (G-1312C), and a DAD detector (G-4212B). The mobile phase consisted of solvent A (H_2_O + 0.1% formic acid) and solvent B (CH_3_CN + 0.1% formic acid), while the stationary phase was constituted by a Luna C 18 (150 × 2 mm, 5 µm) column (Phenomenex). The wavelength was set at 262 nm (Figure S1). The gradient started from 5% of B ending at 100% of B in 20 min, at a flow rate of 0.2 mL/min. The injection volume was 10 µL. An interval of 15 min was used to allow the column to equilibrate before injection of the next samples. Calibration curves were built with the external standard method, correlating the area of the peaks vs. the concentration. The commercial standard of (±)-2-*cis*, 4-*trans*-abscisic acid was used in different concentrations (0.1, 0.25, 0.50, 0.75, 1 mg/mL) to construct the calibration curve (y = 12,984x + 9664 R² = 0.991). The established method was validated in agreement with the International Conference on Harmonisation of Technical Requirements for Registration of Pharmaceuticals for Human Use (ICH) guidance note which describes validation of analytical methods [51]. Both the precision under conditions of repeatability and intermediate precisions were determined by performing either six injections of standard on the same day or six injections of the same standard on different days, respectively. The limit of detection (LOD) and limit of quantification (LOQ) were calculated on the basis of the data of the regression of the analytical curve and corresponding value are reported in Table 2. The *c*,*t*-ABA calibration curve was used to dose *t*,*t*-ABA and unedone as well.

### 3.6. Multivariate Data Analysis

For the untargeted approach, the chromatograms resulting from the LC-ESI/Orbitrap-MS analysis (negative ion mode) were normalised and aligned using MZmine software [42]. Thanks to the use of this toolbox with normalization of the total raw signal, 253 peaks were detected. After exporting the processed data in tabular format (.cvs file), a further statistical analysis of the data matrix was performed with SIMCA P + software 12.0 (Umetrix AB, Umeå, Sweden) using the PCA method. PCA was performed by applying the area of the peak obtained from LC-MS analysis [52,53] and the data were scaled through the application of Pareto scaling. After PCA, PLS was also applied with the SIMCA P + software. PLS is a regression technique used to relate two sets of data. The Y used for the statistical model was the perception of bitterness returned by the different fractions subjected to sensory analysis. Models were validated by cross-validation techniques and permutation tests such as Hotelling’s permutation test and the T^2^ test according to standardised good practice to minimise false discoveries and to obtain robust statistical models. The significance was evaluated by measuring the value of Q^2^, which was higher than 0.5.

## 4. Conclusions

The chemometric model obtained by preliminary PCA of LC-ESI/LTQ-Orbitrap-MS data, followed by a PLS regression model was found to be a selective tool to detect the compounds responsible for the perception of bitterness in ST honey. The study allowed unedone to be identified as the main chemical compound responsible for the bitter taste of strawberry tree honey. Taking into account that unedone was characterised for the first time as a new natural product in strawberry tree honey, it is a novelty for further studies on the action on the bitter taste receptor. Further studies on the 25 human T2Rs bitter taste receptors should be developed to better define the action of unedone. Moreover, investigation of the complex matrix of the ST honey could be interesting due to the presence of the bitter compound unedone, an antagonist for the T2R4 receptor (ABA) and sweets compounds (sugars). Finally, 29 chemical compounds were identified and putatively identified in ST honey, using LC-ESI/LTQ-Orbitrap-MS and LC-ESI/LTQ-Orbitrap-MS/MS, and some of them for the first time in this honey.

## Figures and Tables

**Table 1:** Chemical compounds identified in fractions of strawberry-tree honey by LC-ESI/LTQ-Orbitrap-MS and LC-ESI/LTQ-Orbitrap-MS/MS.

N°	Rt	[M − H]	Molecular Formula	ppm	Identification	MS/MS	Fraction	L.I.	Reference
1	3.97	329.0868	C_14_H_18_O_9_	0.4	glucopiranosyl vanillic acid	167	A	2	[21]
2	6.38	167.0346	C_8_H_8_O_4_	4.3	homogenistic acid	123	A	1	[8]
3	6.86	481.1310	C_22_H_26_O_12_	4.9	arbutin peracetate	271	A	2	[22]
4	10.27	285.1333	C_14_H_22_O_6_	0.1	methacrylic acid, diester with triethylene glycol	-	L	3	[23]
5	11.34	199.0972	C_10_H_16_O_4_	3.7	camphoric acid	155	B, Z	2	[24]
6	13.76	301.1798	C_19_H_26_O_3_	−0.03	allethrin	133	V	2	[25]
7	13.85	275.1280	C_16_H_20_O_4_	0.9	propenoic acid, dimethoxyphenyl-methyl-butenyl ester	71	D	3	[26]
8	14.32	447.1277	C_22_H_24_O_10_	−1.4	sakuranin	285	Z	2	[27]
9	14.62	303.1228	C_17_H_20_O_5_	0.8	(±)-oleocanthal isomer	137/119	F	3	[28]
10	14.71	263.1278	C_15_H_20_O_4_	0.9	(±)-2-cis, 4-*trans*-abscisicacid (*c*,*t*-ABA)	219/204/153	A/B/C	1	[9]
11	15.05	335.1126	C_17_H_20_O_7_	0.3	tutin, 6-acetate	293	Z	3	[29]
12	15.40	153.0922	C_9_H_14_O_2_	4.8	2-hydroxyisophorone	135	A/B/C/D	2	[30]
13	15.44	219.1385	C_14_H_20_O_2_	2.7	di-*tert*-butyl-benzoquinone	107	L	2	[24]
14	15.78	263.1281	C_15_H_20_O_4_	1	(±)-2-*trans*, 4-*trans*-abscisic acid (*t*,*t*-ABA)	219/204/153	A/B/C/D/E/F/H	1	[9]
15	15.83	239.091	C_13_H_20_O_4_	1.2	unedone	151/107	Z	2	[9]
16	15.83	359.1489	C_20_H_24_O_6_	0.29	triptolide	340/329/311	Z	2	[31]
17	16.18	287.1642	C_18_H_24_O_3_	−0.2	estriol	171	V	2	[32]
18	16.39	415.2107	C_24_H_32_O_6_	−1.6	desonide	397	L	2	[33]
19	16.39	201.1280	C_14_H_18_O	3.5	amylcinnamaldehyde	183	A	2	[34]
20	16.39	219.1386	C_14_H_20_O_2_	2.9	di-*tert*-butyl-benzoquinone	107	A	2	[24]
21	16.99	241.1225	C_16_H_18_O_2_	0.9	Bisphenol B	211	P	2	[35]
22	17.04	177.0917	C_11_H_14_O_2_	3.6	4*-tert*-butylbenzoic acid	121	V	2	[36]
23	17.04	417.2269	C_24_H_34_O_6_	−0.9	deoxyphorbol -isobutyrate	347	V	2	[37]
24	19.11	219.1385	C_14_H_20_O_2_	2.4	di-*tert*-butyl-benzoquinone isomer	107	P	2	[24]
25	19.11	415.2110	C_24_H_32_O_6_	−0.8	desonide	397/197	P	2	[33]
26	20.71	325.1438	C_20_H_22_O_4_	0.8	hydroxy-methyl-butenyl-oxyphenyl-ethenyl-methoxyphenol	153	Z	2	[38]
27	21.61	287.2220	C_16_H_32_O_4_	1.1	dihydroxypalmitic acid	147/121/109	Z/V	2	[39]
28	22.60	253.0497	C_15_H_10_O_4_	0.8	chrysin	255/153	Z	2	[40]
29	22.60	437.1952	C_26_H_30_O_6_	−0.7	kurarinone	301	Z	2	[41]

L.I.: Level of identification.

**Table 2:** Compounds quantified by HPLC-DAD at λ = 262 nm (mg/g, *n* = 3).

Sample	Bitter Taste ^a^	*c*,*t*-ABA	*t*,*t*-ABA ^b^	Unedone ^b^
A	0	28.39 ± 1.59	12.36 ± 1.62	nd
B	0	46.14 ± 2.03	91.98 ± 2.05	nd
C	0	11.29 ± 1.05	25.02 ± 1.87	nd
D	1	tr	54.32 ± 2.60	nd
E	1	nd	29.61 ± 1.43	nd
F	2	nd	94.59 ± 1.55	nd
H	3	nd	21.40 ± 1.75	tr
L	3	nd	tr	tr
Z	4	nd	nd	7.10 ± 0.50
LOD (mg/L)		0.4	0.6	0.3
LOQ (mg/L)		1.2	1.9	0.9

^a^ Level of bitter perception: (0) no bitter taste; (1) barely detectable; (2) weak, (3) moderate (4) strong; ^b^ dosed with *c*,*t*-ABA calibration curve; nd: not detected (<LOD); traces (<LOQ).

## Data Availability

The data presented in this study are available in article and supplementary material.

## Supplementary Materials

The following are available online, Table S1. Sensory analysis results (5 judges, mean ± sd) of the bitter perception in the 21 fractions obtained from the fractioning of strawberry tree honey on C18 column. Figure S1. LC-DAD representative chromatograms at λ = 262 nm of fraction B, fraction Z, and pure strawberry tree honey (dilution 1:100 in water *w*/*v*). *t*,*t*-ABA: (±)-2-*trans*, 4-*trans*-abscisic acid; *c*,*t*-ABA: (±)-2-*cis*, 4-*trans*-abscisic acid; U: unedone; HGA: homogentisic acid. Chromatographic conditions are described in the text.

## Sample Availability

Honey sample used for this study is available for limited time from the authors.

## References

[1] Rosa A., Tuberoso C.I.G., Atzeri A., Melis M.P., Bifulco E., Dessì M.A. (2011). Antioxidant profile of strawberry tree honey and its marker homogentisic acid in several models of oxidative stress. Food Chem..

[2] Tuberoso C.I.G., Boban M., Bifulco E., Budimir D., Pirisi F.M. (2013). Antioxidant capacity and vasodilatory properties of Mediterranean food: The case of Cannonau wine, myrtle berries liqueur and strawberry-tree honey. Food Chem..

[3] Afrin S., Forbes-Hernandez T.Y., Gasparrini M., Bompadre S., Quiles J.L., Sanna G., Spano N., Giampieri F., Battino M. (2017). Strawberry-tree honey induces growth inhibition of human colon cancer cells and increases ROS generation: A comparison with Manuka honey. Int. J. Mol. Sci..

[4] Ośes S.M., Nieto S., Rodrigo S., Pérez S., Rojo S., Sancho M.T., Fernández-Muiño M.Á. (2020). Authentication of strawberry tree (*Arbutus unedo* L.) honeys from southern Europe based on compositional parameters and biological activities. Food Biosci..

[5] Di Petrillo A., Santos-Buelga C., Era B., González-Paramás A.M., Tuberoso C., Medda R., Pintus F., Fais A. (2018). Sardinian honeys as sources of xanthine oxidase and tyrosinase inhibitors. Food Sci. Biotechnol..

[6] Jurič A., Gašić U., Brčić Karačonji I., Jurica K., Milojković-Opsenica D. (2020). The phenolic profile of strawberry tree (*Arbutus unedo* L.) honey. J. Serb. Chem. Soc..

[7] Miguel M.G., Faleiro M.L., Guerreiro A.C., Antunes M.D. (2014). Arbutus unedo L.: Chemical and biological properties. Molecules.

[8] Cabras P., Angioni A., Tuberoso C., Floris I., Reniero F., Ghelli S. (1999). Homogentisic acid: A phenolic acid as a marker of strawberry-tree (Arbutus unedo) honey. Food Chem..

[9] Tuberoso C.I.G., Bifulco E., Caboni P., Cottiglia F., Cabras P., Floris I. (2010). Floral markers of strawberry tree (Arbutus unedo L.) honey. J. Agric. Food Chem..

[10] Fierro F., Giorgetti A., Carloni P., Meyerhof W., Alfonso-Prieto M. (2019). Dual binding mode of “bitter sugars” to their human bitter taste receptor target. Sci. Rep..

[11] Izawa K., Amino Y., Kohmura M., Ueda Y., Kuroda M., Liu H.-W., Mander L. (2010). 4.16—Human–Environment Interactions—Taste. Comprehensive Natural Products II.

[12] Meyerhof W., Batram C., Kuhn C., Brockhoff A., Chudoba E., Bufe B., Appendino G., Behrens M. (2010). The molecular receptive ranges of human TAS2R bitter taste receptors. Chem. Senses.

[13] Valdés A., Cifuentes A., León C. (2017). Foodomics evaluation of bioactive compounds in foods. Trends Anal. Chem..

[14] Cerulli A., Masullo M., Montoro P., Hosek J., Pizza C., Piacente S. (2018). Metabolite profiling of “green” extracts of *Corylus avellana* leaves by ^1^H NMR spectroscopy and multivariate statistical analysis. J. Pharm. Biomed. Anal..

[15] La Barbera G., Capriotti A.L., Cavaliere C., Montone C.M., Piovesana S., Samperi R., Zenezini Chiozzi R., Laganà A. (2017). Liquid chromatography-high resolution mass spectrometry for the analysis of phytochemicals in vegetal-derived food and beverages. Food Res. Int..

[16] D’Urso G., d’Aquino L., Pizza C., Montoro P. (2015). Integrated mass spectrometric and multivariate data analysis approaches for the discrimination of organic and conventional strawberry (Fragaria ananassa Duch.) crops. Food Res. Int..

[17] Pascale R., Bianco G., Cataldi T.R.I., Schmitt Kopplin P., Bosco F., Vignola L., Uhl J., Lucio M., Milella L. (2018). Mass spectrometry-based phytochemical screening for hypoglycemic activity of Fagioli di Sarconi beans (Phaseolus vulgaris L.). Food Chem..

[18] D′Urso G., Montoro P., Piacente S. (2020). Detection and comparison of phenolic compounds in different extracts of black currant leaves by liquid chromatography coupled with High-Resolution ESI-LTQ-Orbitrap MS and High-Sensitivity ESI-QTrap MS. J. Pharm. Biomed. Anal..

[19] KNApSAcK Core System. http://www.knapsackfamily.com/knapsack_core/top.php.

[20] Sumner L.W., Amberg A., Barrett D., Beale M.H., Beger R., Daykin C.A., Fan T.W., Fiehn O., Goodacre R., Griffin J.L. (2007). Proposed minimum reporting standards for chemical analysis—Chemical Analysis Working Group (CAWG) Metabolomics Standards Initiative (MSI). Metabolomics.

[21] Pimpao R.C., Dew T., Figueira M.E., McDougall G.J., Stewart D., Ferreira R.B., Santos C.N., Williamson G. (2014). Urinary metabolite profiling identifies novel colonic metabolites and conjugates of phenolics in healthy volunteers. Mol. Nutr. Food Res..

[22] Karikas G.A., Giannitsaros A. (1990). Phenolic glucosides from leaves of Arbutus unedo. Plantes Med. Phytothe..

[23] Marmarinos V., Paschalakis P. (2012). Photopolymerization Material for Gums Isolation. U.S. Patent.

[24] Ouchemoukh S., Amessis-Ouchemoukh N., Gomez-Romero M., Aboud F., Giuseppe A., Fernandez-Gutierrez A., Segura-Carretero A. (2017). Characterisation of phenolic compounds in Algerian honeys by RP-HPLC coupled to electrospray time-of-flight mass spectrometry. LWT—Food Sci. Technol..

[25] Long E.Y., Krupke C.H. (2016). Non-cultivated plants present a season-long route of pesticide exposure for honey bees. Nat. Commun..

[26] Wilkins A.L., Lu Y., Tan S.T. (1993). Extractives from New Zealand honeys. 4. Linalool derivatives and other components from nodding thistle (Carduus nutans) honey. J. Agric. Food Chem..

[27] Kaskoniene V., Maruska A., Kornysova O., Charczun N., Ligor M., Buszewski B. (2009). Quantitative and qualitative determination of phenolic compounds in honey. Chem. Technol..

[28] Rouphael Y., Bernardi J., Cardarelli M., Bernardo L., Kane D., Colla G., Lucini L. (2016). Phenolic compounds and sesquiterpene lactones profile in leaves of nineteen artichoke cultivars. J. Agric. Food Chem..

[29] Blunt J., Munro M.G., Swallow W. (1979). Carbon-13 NMR analysis of tutin and related substances: Application to the identification of minor components of toxic honey. Aust. J. Chem..

[30] Dalla Serra A., Franco M.A., Mattivi F., Ramponi M., Vacca V., Versini G. (1999). Aroma characterization of Sardinian strawberry tree (Arbutus unedo L.) honey. Ital. J. Food Sci..

[31] Sun M., Zhao L., Wang K., Han L., Shan J., Wu L., Xue X. (2019). Rapid identification of “mad honey” from Tripterygium wilfordii Hook. f. and Macleaya cordata (Willd) R. Br using UHPLC/Q-TOF-MS. Food Chem..

[32] Ma L., Ashworth D., Yates S.R. (2016). Simultaneous determination of estrogens and progestogens in honey using high performance liquid chromatography-tandem mass spectrometry. J. Pharm. Biomed..

[33] Staub Spörri A., Jan P., Cognard E., Ortelli D., Edder P. (2014). Comprehensive screening of veterinary drugs in honey by ultra-high-performance liquid chromatography coupled to mass spectrometry. Food Addit. Contam. A.

[34] Bentivenga G., D’Auria M., Fedeli P., Mauriello G., Racioppi R. (2004). SPME-GC-MS analysis of volatile organic compounds in honey from Basilicata. Evidence for the presence of pollutants from anthropogenic activities. Int. J. Food Sci..

[35] Cešen M., Lambropoulou D., Laimou-Geraniou M., Kosjek T., Blaznik U., Heath D., Heath E. (2016). Determination of bisphenols and related compounds in honey and their migration from selected food contact materials. J. Agric. Food Chem..

[36] Rodríguez-Gonzalo E., Domínguez-Alvarez J., García-Gómez D., García-Jiménez M.G., Carabias-Martínez R. (2010). Determination of endocrine disruptors in honey by CZE-MS using restricted access materials for matrix cleanup. Electrophoresis.

[37] Sosath S., Ott H.H., Hecker E. (1988). Irritant principles of the spurge family (Euphorbiaceae). XIII. Oligocyclic and macrocyclic diterpene esters from latexes of some *Euphorbia* species utilized as source plants of honey. J. Nat. Prod..

[38] Duke C.C., Tran V.H., Duke R.K., Abu-Mellal A., Plunkett G.T., King D.I., Hamid K., Wilson K.L., Barrett R.L., Bruhl J.J. (2017). A sedge plant as the source of Kangaroo Island propolis rich in prenylated *p*-coumarate ester and stilbenes. Phytochemistry.

[39] Leyva-Jimenez F.J., Lozano-Sanchez J., Borras-Linares I., Cadiz-Gurrea M.d.l.L., Mahmoodi-Khaledi E. (2019). Potential antimicrobial activity of honey phenolic compounds against Gram positive and Gram negative bacteria. LWT—Food Sci. Technol..

[40] Seraglio S.K.T., Valese A.C., Daguer H., Bergamo G., Azevedo M.S., Gonzaga L.V., Fett R., Costa A.C.O. (2016). Development and validation of a LC-ESI-MS/MS method for the determination of phenolic compounds in honeydew honeys with the diluted-and-shoot approach. Food Res. Int..

[41] Liu P., Deng T., Ye C., Qin Z., Hou X., Wang J. (2009). Identification of kurarinone by LC/MS and investigation of its thermal stability. J. Chil. Chem. Soc..

[42] MZmine 2. http://mzmine.github.io/.

[43] Schievano E., Stocchero M., Morelato E., Facchin C., Mammi S. (2012). An NMR-based metabolomic approach to identify the botanical origin of honey. Metabolomics.

[44] Stocchero M. (2020). Relevant and irrelevant predictors in PLS2. J. Chemom..

[45] Pydi S.P., Jaggupilli A., Nelson K.M., Abrams S.R., Bhullar R.P., Loewen M.C., Chelikani P. (2015). Abscisic acid acts as a blocker of the bitter taste G protein-coupled receptor T2R4. Biochemistry.

[46] Remya C., Dileep K.V., Tintu I., Variyar E.J., Sadasivan C. (2014). Flavanone glycosides as acetylcholinesterase inhibitors: Computational and experimental evidence. Indian J. Pharm. Sci..

[47] Yang J., Chen H., Wang Q., Deng S., Huang M., Ma X., Song P., Du J., Huang Y., Wen Y. (2018). Inhibitory effect of kurarinone on growth of human non-small cell lung cancer: An experimental study both in vitro and in vivo studies. Front. Pharm..

[48] Huai D. (2014). Anti-fibrotic effect of kurarinone in treatment of chronic hepatitis B. Xiandai Zhenduan Yu Zhiliao.

[49] Deiana V., Tuberoso C., Satta A., Pinna C., Camarda I., Spano N., Ciulu M., Floris I. (2016). Relationship between markers of botanical origin in nectar and honey of the strawberry tree (Arbutus unedo) throughout flowering periods in different years and in different geographical areas. J. Apic. Res..

[50] D′Urso G., Maldini M., Pintore G., d′Aquino L., Montoro P., Pizza C. (2016). Characterisation of Fragaria vesca fruit from Italy following a metabolomics approach through integrated mass spectrometry techniques. LWT—Food Sci. Technol..

[51] EMEA Quality Guidelines: Validation of Analytical Procedures: Text and Methodology (ICH Q2). http://www.emea.europa.eu/pdfs/human/ich/038195en.pdf.

[52] Mari A., Montoro P., Pizza C., Piacente S. (2012). Liquid chromatography tandem mass spectrometry determination of chemical markers and principal component analysis of Vitex agnus-castus L. fruits (Verbenaceae) and derived food supplements. J. Pharm. Biomed. Anal.

[53] Soufi S., D′Urso G., Pizza C., Rezgui S., Bettaieb T., Montoro P. (2016). Steviol glycosides targeted analysis in leaves of Stevia rebaudiana (Bertoni) from plants cultivated under chilling stress conditions. Food Chem..

