# Supplementary material for: LC-ESI/LTQ-Orbitrap-MS Based Metabolomics in Evaluation of Bitter Taste of Arbutus unedo Honey
Source: Molecules. 2021 May 8;26(9):2765. doi: 10.3390/molecules26092765 (PMC8125850; doi:10.3390/molecules26092765)
Supplement: Supplementary file 1 [file molecules-26-02765-s001.zip › molecules-1173743-supplementary.pdf]

molecules

Article

LC-ESI/LTQ-Orbitrap-MS Based Metabolomics in Evaluation of Bitter Taste of *Arbutus unedo* Honey

Supporting information

Paola Montoro [1], Gilda D'Urso [1], Adam Kowalczyk[2] and Carlo Ignazio Giovanni Tuberoso [3,*]

[1] Department of Pharmacy, University of Salerno, via Giovanni Paolo II, 132, 84084 Fisciano (SA), Italy; pmontoro@unisa.it (P.M.); gidurso@unisa.it (G.D'U.)

[2] Department of Pharmacognosy and Herbal Medicines, Wrocław Medical University, ul. Borowska, 211, 50-556 Wrocław, Poland; adam.kowalczyk@umed.wroc.pl

[3] Department of Life and Environmental Sciences, University of Cagliari, University Campus, S.P. Monserrato-Sestu km 0.700 - 09042 Monserrato (CA), Italy; tuberoso@unica.it

* Correspondence: tuberoso@unica.it; Tel.: +39-070-675-8644

Table of Contents

Table S1. Sensory analysis results (5 judges, mean ± sd) of the bitter perception in the 21 fractions obtained from the fractioning of strawberry tree honey on C18 column.
Figure S1. LC-DAD representative chromatograms at λ = 262 nm of fraction B, fraction Z, and pure strawberry tree honey (dilution 1:100 in water w/v). *t*,*t*-ABA: (±)-2-*trans*,4-*trans*-abscisic acid; *c*,*t*-ABA: (±)-2-*cis*,4-*trans*-abscisic acid; U: unedone; HGA: homogentisic acid. Chromatographic conditions are described in the text.

Table S1. Sensory analysis results (5 judges, mean ± sd) of the bitter perception in the 21 fractions obtained from the fractioning of strawberry tree honey on C18 column.

Fraction	Code	Level of bitter perception[a]						
		J#1	J#2	J#3	J#4	J#5	Mean	± sd
1	A	0	0	0	0	0	0	0
2	B	0	0	0	0	0	0	0
3	C	1	0	0	0	0	0	0
4	D	1	1	0	1	1	1	0
5	E	1	1	1	1	1	1	0
6	F	2	2	2	2	2	2	0
7	G	3	2	3	3	3	3	0
8	H	3	3	3	3	3	3	0
9	I	3	3	3	2	3	3	0
10	L	3	3	3	3	3	3	0
11	M	2	1	1	1	1	1	0
12	N	1	1	1	1	1	1	0
13	O	1	1	1	1	2	1	0
14	P	2	3	2	3	3	3	1
15	Q	1	1	1	2	1	1	0
16	R	1	1	1	1	1	1	0
17	S	1	1	1	1	1	1	0
18	T	1	1	1	1	1	1	0
19	U	1	1	1	1	1	1	0
20	V	2	2	2	3	2	2	0
21	Z	4	4	4	4	4	4	0

[a] Level of bitter perception: 0) no bitter taste; 1) barely detectable; 2) weak, 3) moderate 4) strong.

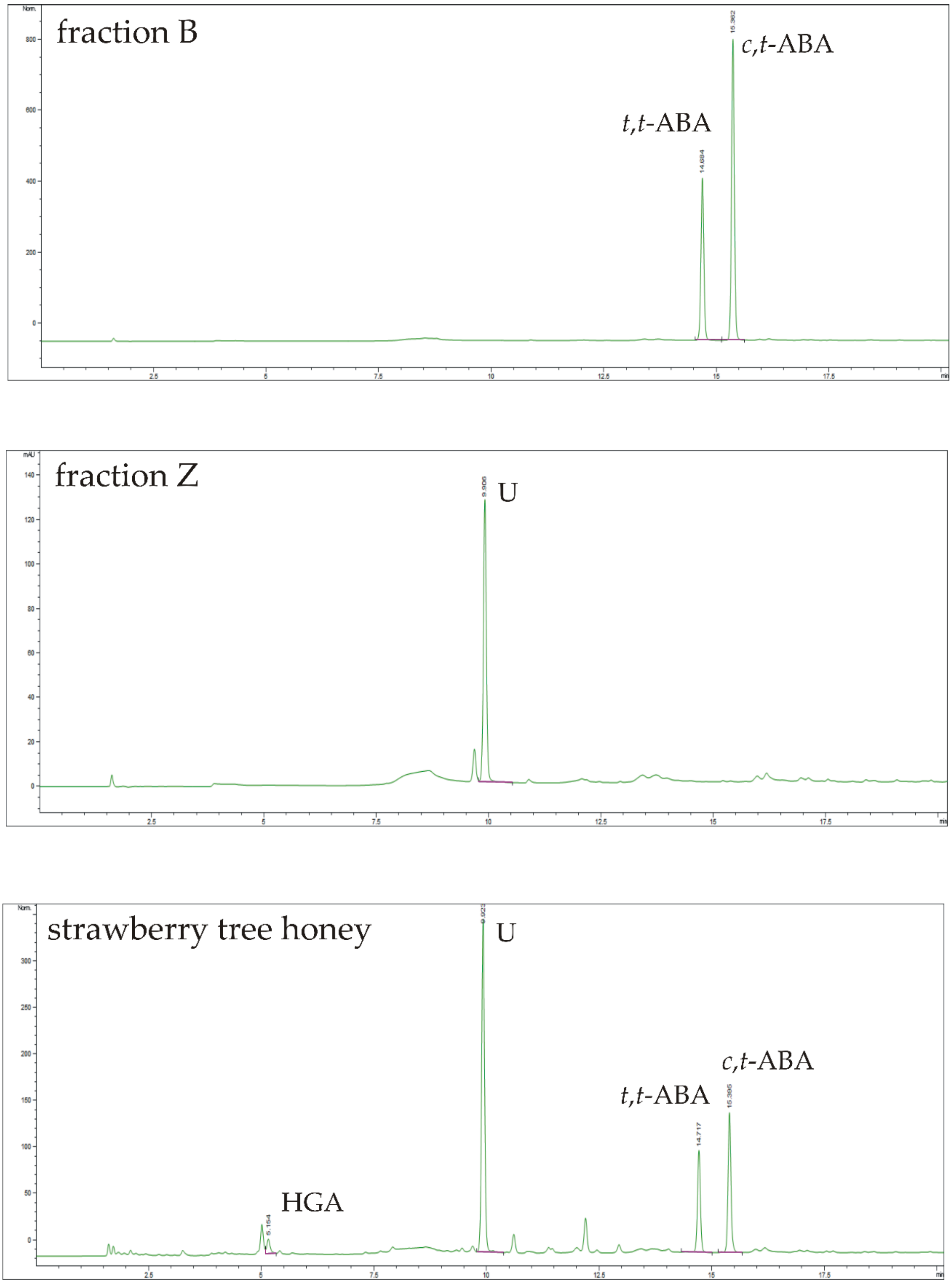

Figure S1. LC-DAD representative chromatograms at $\lambda = 262$ nm of fraction B, fraction Z, and pure strawberry tree honey (dilution 1:100 in water w/v). *t,t*-ABA: (±)-2-*trans*,4-*trans*-abscisic acid; *c,t*-ABA: (±)-2-*cis*,4-*trans*-abscisic acid; U: unedone; HGA: homogentisic acid. Chromatographic conditions are described in the text.
